# Black soldier fly larvae used for environmental enrichment purposes: Can they affect the growth, slaughter performance, and blood chemistry of medium-growing chickens?

**DOI:** 10.3389/fvets.2022.1064017

**Published:** 2022-12-14

**Authors:** Valentina Bongiorno, Marta Gariglio, Valeria Zambotto, Eleonora Erika Cappone, Ilaria Biasato, Manuela Renna, Claudio Forte, Carl Coudron, Stefania Bergagna, Francesco Gai, Achille Schiavone

**Affiliations:** ^1^Department of Veterinary Sciences, University of Turin, Turin, Italy; ^2^National Research Council, Institute of Sciences of Food Production, Turin, Italy; ^3^Department of Agricultural, Forest and Food Sciences, University of Turin, Turin, Italy; ^4^Provincial Research and Advice Centre for Agriculture and Horticulture (Inagro vzw), Roeselare-Beitem, Belgium; ^5^Istituto Zooprofilattico Sperimentale del Piemonte, Liguria e Valle d'Aosta, Turin, Italy

**Keywords:** organic rearing, live larvae, free-range chickens, larva consumption duration, animal welfare

## Abstract

**Introduction:**

This research has been aimed at evaluating the effects of live black soldier fly larvae (BSFL) (*Hermetia illucens*) on the growth, slaughtering performance, and blood parameters of medium-growing chickens.

**Methods:**

A total of 240, 28-day-old, Label Rouge Naked Neck chickens were allotted to four experimental groups, according to the gender (males-females) and to the absence (control group, C) or presence (larvae group, L) of a dietary supplementation with 10% live BSFL, on the basis of the expected average daily feed intake (ADFI) (6 replicates/diet, 10 chickens/replicate). The birds were weighed weekly, and the feed consumption was recorded to calculate the average live weight, feed conversion ratio (FCR), average daily gain (ADG), and the ADFI. At 82 days of age, 2 birds/replicate (12 birds/diet) were selected and slaughtered. The blood samples were collected, and the carcass traits (carcass, breast, thigh, and organ weights and yields) were assessed.

**Results and discussions:**

Overall, the administered live BSFL did not impair the growth and slaughtering performance, or the blood traits, while the C females showed a better FCR than the treated ones (*P* < 0.05). The live BSFL consumption time was longer for the females than for the males (*P* < 0.001). The weight of the immune organs (spleen and bursa of Fabricius) increased as the live BSFL supplementation increased (*P* < 0.05). Furthermore, the provision of live BSFL reduced the gamma glutamyl transferase (GGT, U/l) activity content in the blood (*P* < 0.05). Finally, both the leukocytes (%) and the monocytes (%) were more abundant in the C groups than in the larvae ones (*P* < 0.05 and *P* < 0.01, respectively). In short, the supplementation of live BSFL can be used successfully as an environmental enrichment, without affecting the growth performance of male birds. Furthermore, the immune organ activity could be enhanced by the provision of live BSFL.

## Introduction

It is well known that organic and free-range rearing systems for poultry are perceived by consumers to provide sustainable products, thereby guaranteeing environmental protection and animal welfare (1). These aspects also represent fundamental factors that can affect the choices of consumers and can orient their purchasing decisions (2–4). Benefits can hence be introduced for the farmer who, by guaranteeing an increase in animal welfare, can obtain significant economic profits. It has been demonstrated that the welfare status of broilers can influence their performance (5). The use of insects in chicken diets could be a valid tool, because of their characteristics, to support and promote bird welfare. Indeed, live insects can be considered as an environmental enrichment, as they are able to stimulate the curiosity of poultry as a result of their motility (6). Moreover, it is known that the quality of the substrate in terms of visual, tactile and gustatory appreciation, affects the interest of birds. Hence, the larvae's abundance in moisture and fat could be an interesting feed source for poultry, useful to balance their nutrient intake (6, 7). Biasato et al. (8) have recently reported an improvement in the welfare of broilers fed diets supplemented with 5% yellow mealworm (*Tenebrio molitor*) or 5% live black soldier fly larvae (*Hermetia illucens*) (BSFL), as well as an increase in physical activity. Furthermore, insects are a natural feed source for poultry, and are capable of promoting their foraging behavior, as they capture their attention and reduce the poultry aggressiveness toward conspecifics (6, 9). Finally, insects can minimize and valorize food waste, which is a promising substrate for insect feeding (6, 10–12). Nevertheless, the provision of live larvae to poultry has been poorly investigated. The few published papers about the use of live insect larvae in poultry nutrition have mainly focused on the provision of live black soldier fly larvae to turkeys (13), broiler chickens (14, 15), and laying hens (16, 17), and have evaluated the effects on the growth, health status, and slaughtering performance of the birds. Veldkamp and van Niekerk (13) revealed a higher daily feed intake (DFI) and body weight gain in turkeys fed 10% live BSFL (on an as fed basis) than control groups. The physical exertion of broilers, as well as their hock burn score, can be improved by the administration of live larvae (supplemented as fed of 5 or 10% live BSFL, twice or four times/day), as reported by Ipema et al. (14). Dietary supplementation with live yellow mealworm (5% as fed) further improved the feed conversion ratio (FCR) of broilers in a trial conducted by Bellezza Oddon et al. (15). Finally, a lower feed consumption and a higher live body weight increase were reported in laying hens fed live BSFL *ad libitum* than in control birds and groups with controlled larvae provision (10% or 20% as fed) (17). In addition to these parameters, the recording of the larvae consumption duration could be useful, and this method had already been experimented by Veldkamp and van Niekerk (13) and Bellezza Oddon et al. (15) to evaluate the appreciation of larvae by birds, as well as to monitor their level of confidence in larvae consumption over time. Moreover, the effect of insect provision could be reflected in the blood traits, which is an important animal welfare indicator (18). A previous study reported no negative effects on blood traits of broiler chickens supplemented with live BSFL and yellow mealworm larvae (15), while a reduction in triglycerides and in the cholesterol levels was observed in the blood of Muscovy ducks fed increasing levels (0, 3, 6, and 9%) of black soldier fly meal (16).

The aim of this research was to provide novel insights into the effects of the provision of live BSFL in free-range and organic systems, since chickens intended for meat consumption have never been tested before from a long-term rearing perspective (considering the classic broiler rearing cycle). Thus, the growth and slaughtering performance, the larvae consumption duration, and the blood parameters of the birds were investigated in relation to this kind of supplementation.

## Materials and methods

### Animals and husbandry

A total of 300 1-day-old Label Naked Neck birds (Hubbard JA57 hybrid) were purchased from a commercial hatchery (sexed chicks, sex ratio 1:1) and transferred to the poultry facility of the University of Turin (Department of Agricultural, Forest and Food Sciences) (North-West Italy), where the trial was carried out (coordinates latitude: 44.88572, longitude: 7.68381, altitude: 239 mamsl). The experiment was previously approved by the Bioethical Committee of the University of Turin (Italy; Prot. No. 814715). The birds were vaccinated against coccidiosis, and Newcastle and Marek's diseases. The chicks were reared from 1 to 21 days of age in an environmentally controlled poultry house and distributed according to gender in four 3.4 m × 2.9 m pens with rice hulls as bedding. Each pen was equipped with 3 feeders and 3 drinkers, and automatic ventilation and illumination systems were available. The adopted lighting schedule was 23L:1D during the first 3 days, according to the Hubbard guidelines (19). Moreover, infrared lamps were at the disposal of the birds for the first 2 weeks of age. Subsequently, the photoperiod was gradually modulated to recreate, as much as possible, the natural environmental conditions of the experimental building in which the birds were to be housed at 21 days of age (12L:12D). At that moment, the birds were individually weighed and tagged with a wing mark. Then 120 males (M) and 120 females (F) were selected, on a uniform live weight (LW, g) basis, and allotted to 24 pens. The 2.2 × 3.5 m pens (with rice husk as bedding) each had the possibility of outdoor access (2.2 × 4.5 m), which was ensured for all the birds from 49 days of age to the end of the experiment (82 days of age). All the birds were subjected to the same management and environmental conditions in respect of the European Union's regulations on organic farming (20). Natural ventilation and photoperiods (from 12L:12D in October 2021 to 10L:14D at the end of November 2021) were applied for the entire duration of the trial. The average temperature was 12.8°C (min 5°C; max 22°C) in October 2021 and 7.6°C (min −1°C; max 16°C) in November 2021. The mortality and health status of the birds were checked and recorded daily. Regarding the experimental design, four experimental groups were created on d 21 according to the gender and the diet (10 chicken/pen, 6 replicates/diet):

1. Control males (CM): fed a basal organic feed.
2. Control females (CF): fed a basal organic feed.
3. Larvae males (LM): fed a basal organic feed, supplemented with 10% live BSFL (as fed, 33.63/100g on dry matter–DM) on the basis of the expected DFI.
4. Larvae females (LF): fed a basal organic feed, supplemented with 10% live BSFL (as fed, 33.63/100g DM) on the basis of the expected DFI.

The expected DFI of the birds was taken from the Hubbard guidelines (19). The birds always had free access to water and feeds. A starter diet was adopted until d 28 (22.92/100g of crude protein (CP), 15.36 MJ/kg of apparent metabolizable energy) and a grower feed was then provided from d 28 to d 82 (20.55/100g of crude protein, 14.19 MJ/kg of apparent metabolizable energy) (Verzuolo Biomangimi s.r.l.—Verzuolo, CN, Italy). The feed was analyzed, and the DM (method number 934.01), ash (method number 942.05), and ether extract (EE, method number 2003.05) were calculated [DM, ash; EE: (21)]. The CP was determined by means of the Dumas method (22, 23). The feed composition of the feeds is reported in the Table 1. Provision of live larvae was started on d 28, after one-week of adaptation of the birds to the new environment.

**Table 1:** Ingredients and the analyzed chemical composition of the experimental diets^a^.

**Ingredients**	**Starter period (1–28 days)**	**Grower period (28–81 days)**
Corn meal	43.00	50.48
Soybean meal	28.60	16.26
Sunflower meal	10.00	14.46
Corn gluten meal	3.50	4.70
Pea beans	7.00	8.00
Alfalfa meal	2.00	2.50
Soybean oil	1.00	-
Dicalcium phosphate	1.70	0.85
Calcium carbonate	1.55	1.25
Sodium bicarbonate	0.10	-
Mineral-vitamin premix^b^	1.50	1.50
PoultryStar^®^^c^	0.05	-
Chemical composition (g/100 g on an as fed basis)		
DM	90.44	90.61
CP	22.92	20.55
EE	6.19	5.02
CF	5.85	6.26
Ash	7.81	5.65
AMEn^d^, MJ/kg	15.36	14.19
Mineral composition		
Ca	1.27	0.96
P	0.64	0.49
Na	0.18	0.15
Aminoacids		
Methionine	0.34	0.33
Lysine	1.04	0.83

DM, dry matter; CP, crude protein; EE, ether extract; CF, crude fiber; AME, gross energy.

a Values are reported as the mean value of duplicated analyses.

b Nutritional additives: Vitamin A 8.001.60 UI, Vitamin D3 3.000.60 UI, Betaine anhydrous 600.48 mg, Biotin 0.04 mg, Choline chloride 333.07 mg, Folic acid 0.81 mg, Niacinamide 25.01 mg, Calcium pantothenate 7.28 mg, Vitamin B1 0.75 mg, Vitamin B12 0.02 mg, Vitamin B6 1.60 mg, Vitamin E 18.50 mg, Vitamin K3 2.50 mg, Copper (Copper-II sulfate pentahydrate) 10.00 mg, Iodine (Calcium iodate anhydrous) 1.50 mg, Iron (Iron-II sulfate monohydrate) 44.01 mg, Manganese (Manganese-II oxide) 62.01 mg, Selenium (Sodium Selenite) 0.25 mg, Zinc (Zinc sulfate monohydrate) 50.01 mg. Zootechnical additives: 4a1604i Endo-1,3(4)-beta-glucanase EC 3.2.1.6 1.500,30 UV, 4a1604i Endo-1,4-beta-xylanase EC 3.2.1.8 1.100,22 UV, Technological additives: E 562 Sepiolite 224.32 mg, 1m558 Bentonite 0.54 mg.

c Poultrystar^®^: prebiotic and probiotic complex produced by Koninklijke DSM N.V., Heerlen, The Netherlands.

d Calculated according to INRA (24).

### Larvae management and chemical analyses

The live BSFL were provided by INAGRO (Ieperseweg 87, 8800 Rumbeke-Beitem–Belgium). The larvae were shipped weekly in an insulated container with cool bags to keep them chilled and to avoid their death during the 24-h journey. Once the larvae had arrived at the poultry experimental facility, they were stored at 16°C in a climatic chamber to induce the diapause of insects and fix the larvae instar for the entire week (25). A total of 100 g of BSFL samples were collected each week and stored at −80°C and subsequently analyzed for DM (method number 934.01), ash (method number 942.05), and EE (method number 2003.05) [DM, ash; EE: (21)]. The CP and the chitin contents were determined by means of the Dumas and Finke methods, respectively (22, 23, 26). The gross energy (GE) content was obtained by means of an adiabatic calorimetric bomb (C000; IKA). Finally, an analysis of the aminoacidic (AA) composition was performed according to the method used by Hewitson et al. (27). The average composition of the BSFL is reported in the Table 2.

**Table 2:** The analyzed proximate and amino acid composition of black soldier fly larvae.

**Proximate composition, g/100 g on an as fed basis**	**Values^a^**
DM	33.63
CP	14.39
EE	9.56
Ash	4.34
Chitin	2.00
GE, MJ/kg	8.69
Amino acid, g/100 g on an as fed basis	
Alanine	1.01
Arginine	0.71
Aspartic acid	1.28
Cysteine	0.17
Glutamic acid	1.65
Glycine	0.77
Hystidine	0.44
Isoleucine	0.61
Leucine	0.94
Lysine	0.94
Methionine	0.27
Phenylalanine	0.57
Proline	0.77
Serine	0.64
Threonine	0.54
Tryptophane	2.02
Tyrosine	0.87
Valine	0.81

DM, dry matter; CP, crude protein; EE, ether extract; GE, gross energy.

a Values are reported as the mean value of duplicated analyses.

Prior to being administered to the chickens, the live BSFL were reactivated by heating them at 28°- 30°C for 10 min, in accordance with what was reported by Bellezza Oddon et al. (15). The live BSFL were provided daily at 11.00 a.m., except on Sundays. The larvae consumption duration was recorded daily using a stopwatch that started when a plate was placed in the pen and ended when the plate was empty. The duration of the BSLF consumption was monitored throughout the experimental period considering five recording periods, composed of 10 days each, except for the last one, which only lasted 7 days. The five-recording periods were defined as follows: T1: 28–39 d, T2: 40–50 d, T3: 51–62 d, T4: 63–74 d, and T5: 75–81 d. The larvae were distributed in each pen on two plates (18.8 linear cm and 141.3 cm^2^/chick, Ø 30 cm). Two empty plates were also introduced into the C bird pens.

### Growth performance

The LW (g) and the feed consumption (g) were recorded weekly (electronic scales; KERN PLE-N v. 2.2; KERN & Sohn GmbH; d: 0.1) on a pen basis for the whole experimental period (28–81 d). The average daily gain (ADG, g/d), the average daily feed intake (ADFI, g/d), and the FCR (g/g, DM basis) were calculated both on a weekly basis and for the overall period (28–81 d) on a pen basis. The ADFI was calculated on an as fed basis without considering the larvae administration. However, the average amount of provided BSFL (on an as fed basis, g) was determined. Finally, the DM of the larvae was analyzed (33.63/100 g) and used to adjust the FCR of the birds, according to the method used by Veldkamp and van Niekerk (13) and by Bellezza Oddon et al. (15).

### Slaughtering performance

A total of 48 birds (two birds/pen, 12 birds/diet) were selected on d 82 on the basis of the average LW of the pen and labeled with a shank ring. The individual slaughtering weight (SW, g) was recorded. The birds were slaughtered in a commercial abattoir and blood samples were collected during bleeding. The weight of the ready-to-cook carcass (RTCC, g) (plucked carcasses without feet, neck, head, and organs) was registered. The absolute weight of the heart, spleen, bursa of Fabricius, liver, and abdominal fat was recorded, and the relative weights were calculated as a percentage of the SW. After 24 h of refrigeration (4°C), the chilled carcass (CC, g) weight was registered, and the weight reported as a % of SW. The RTCC yield (%SW), CC yield (%SW), breast yields (%CC weight) and thigh yields (%CC weight) were also calculated.

### Blood analyses

Blood samples were collected from the jugular vein of the 48 selected birds at slaughtering. A total of 2.5 mL was placed in a K_3_EDTA tube and 2.5 mL in a serum-separating tube to evaluate the hematological parameters. A blood smear was prepared from a drop of blood without anticoagulant. A glass slide was used for each bird. The smears were stained using May-Grünwald and Giemsa stains. The total red (erythrocytes, 10^6^, cell/μL) and white (leukocytes, 10^3^, cell/μL) blood cell counts were determined in an improved Neubauer hemocytometer on blood samples previously treated with a 1:200 Natt-Herrick solution (28). One hundred leukocytes, including granular (heterophils, eosinophils, and basophils) and non-granular (lymphocytes and monocytes) leukocytes, were counted on the slide and expressed as a percentage of the total leukocytes, according to Campbell (29). Samples from tubes without any anticoagulant had previously been centrifuged at 3,500 rpm for 15 min at 20°C, after having been left at room temperature for 2 h to favor blood clot formation. The concentrations of alanine aminotransferase (ALT, U/L), aspartate aminotransferase (AST, U/L), creatinine (CRE, mg/dL), total proteins (g/dL), uric acid (mg/dL), cholesterol (mg/dL), triglycerides (mg/dL), gamma glutamyl transferase (GGT, U/L), phosphorus (P, mg/dL), iron (Fe, μg/dL), and magnesium (Mg, mg/dL) were measured using a compact liquid chemistry analyzer system (BT 1500 vet–Futurlab, Padua, Italy).

### Statistical analysis

The data were analyzed by means of IBM SPSS Statistics software, V20.0.0 (IBM). The homogeneity of variance was established by means of Levene's test, and the normality or non-normality of residuals was assessed by means of Shapiro-Wilk's test. The pen was considered as the experimental unit for the larvae consumption duration and growth performance (n=6 per diet), while the animal was used as the experimental unit for the slaughtering performance and the blood parameters (n=12 per diet). A general linear mixed model (GLMM), with a gamma probability distribution and log-link function, was performed to analyze the larvae consumption duration, where the time (i.e., the above mentioned 5 recording periods, namely T1–T5), gender and their interaction were considered as fixed effects, as evaluated by pairwise comparisons, and the replicate was included as repeated measurements on the same pen. A general linear model (GLM) was used for the growth and slaughtering performances and for the blood analyses. The gender, diet, and the interaction between gender and diet were considered by means of a pairwise comparison. *P* ≤ 0.05 were declared as statistically significant, while a statistical trend was defined for *P* ≤ 0.10.

## Results

### Growth performance

The health status of the birds was checked daily, and no mortality was recorded throughout the whole experiment. The growth performance of the birds is summarized in Table 3. A statistical analysis, based on the weekly measurements, was performed, and the complete table is reported in the Supplementary Table S1.

**Table 3:** The growth performance of male and female Label Rouge Naked Neck birds fed a diet supplemented with 10% live black soldier fly larvae; supplementation based on the expected daily feed intake (28–81 d; *n* = 6).

**Item**	**Days**	**Diet (D)**		**Gender (G)**		**SEM**			***P-*** **value**		
		**BSFL**	**Control**	**Male**	**Female**	**D**	**G**	**D × G**	**D**	**G**	**D × G**
LW, g	28	479	475	515	439	2.07	2.07	2.92	0.186	<0.001	0.298
	81	2,372	2,340	2,742	1,970	11.40	11.40	16.12	0.047	<0.001	0.281
ADG, g/d	28–81	35.6	35.2	41.8	28.9	0.24	0.24	0.34	0.229	<0.001	0.700
ADFI, g/d	28–81	111 + 9.93^*^	112	125	98.6	1.16	1.16	1.65	0.572	<0.001	0.152
FCR, g/g	28–81	2.93	2.92	2.74	3.11	0.02	0.02	0.03	0.722	<0.001	0.002

LW, live weight; ADG, average daily gain; ADFI, average daily feed intake (on an as fed basis).

* Indicates the average amount of larvae provided (on an as fed basis, average DM: 33.63/100 g).

FCR, feed conversion ratio (on a dry matter basis, including the larvae intake); BSFL, black soldier fly larvae; SEM, standard error of the mean.

**Figure 1:**
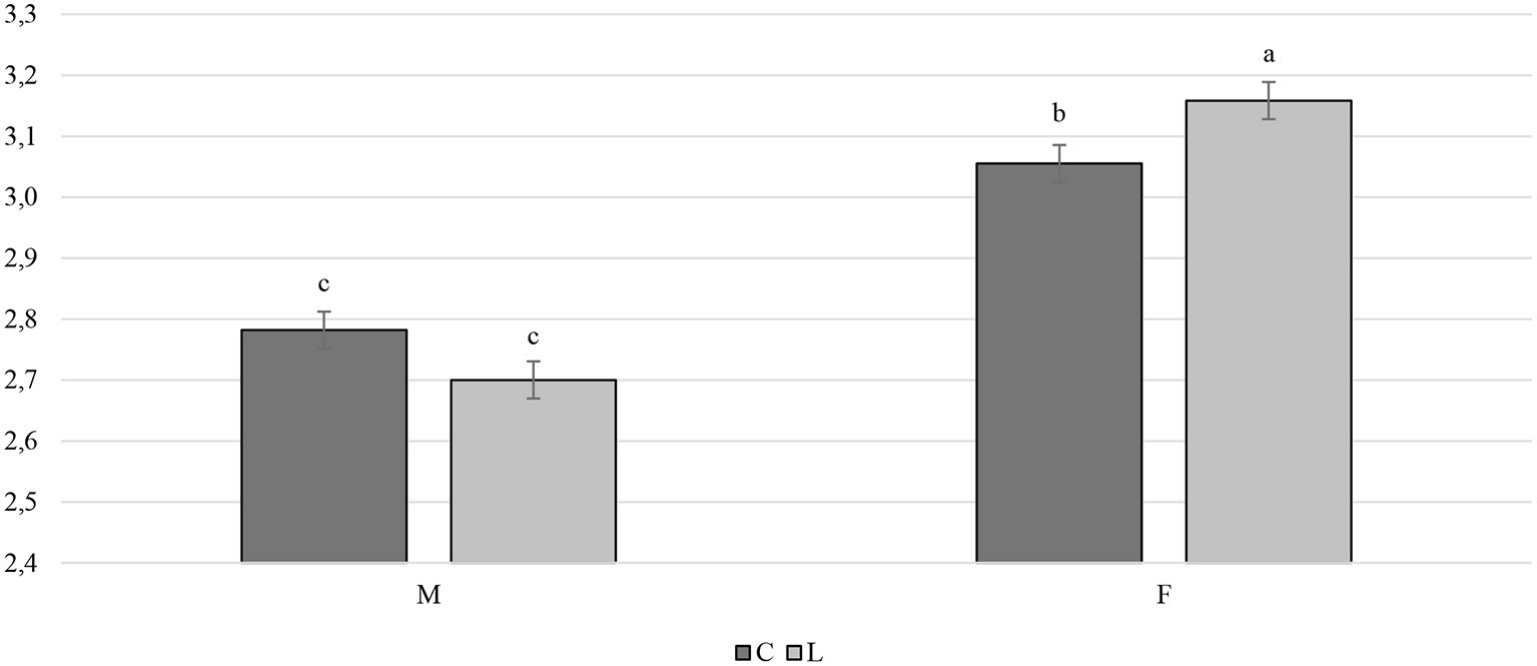
Interaction effect (gender × diet) on the feed conversion ratio of the Label Rouge Naked Neck birds fed a diet supplemented with 10% live black soldier fly larvae; supplementation based on the expected daily feed intake, (28–81 d; *n* = 6). a, b indicate significant differences at *P* < 0.05; F, females; M, males; C, control; L, larvae.

**Figure 2:**
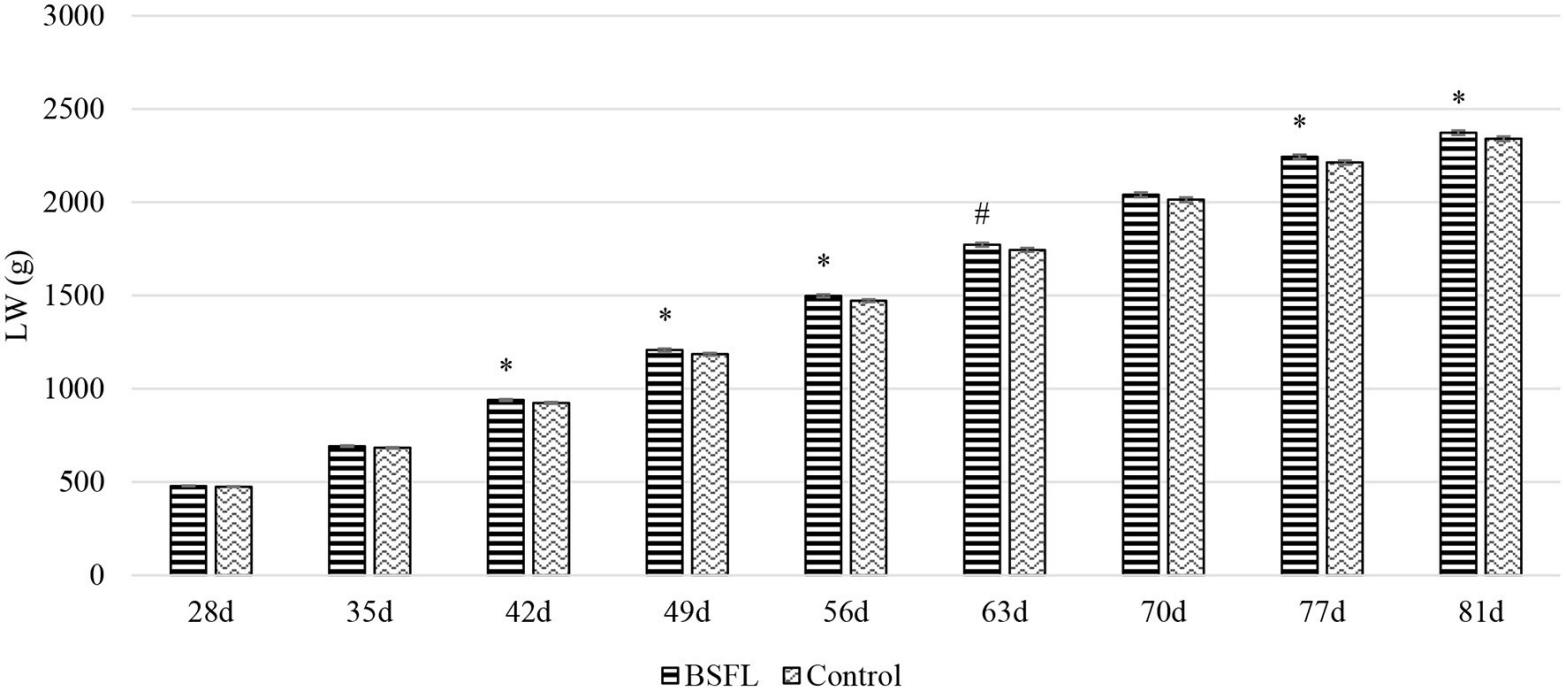
The growth curves of the Label Rouge Naked Neck birds fed a diet supplemented with 10% live black soldier fly larvae; supplementation based on the expected daily feed intake, (28–81 d; *n* = 6). ^#^Indicates a statistical trend for the control and BSFL supplemented birds (*P* ≤ 0.10); *Indicates a statistical difference between the control and BSFL supplemented birds (*P* ≤ 0.05). LW, live weight.

Overall, the larvae supplementation did not affect the growth performance of the birds, apart from the LW at 81 days of age, when the treated birds were heavier (+32.12 g) than the C ones (*P* < 0.05). Instead, the gender always had a significant effect on the growth performance parameters. In other words, the M displayed a higher LW, ADG, and ADFI than the F, as well as a better FCR (*P* < 0.001) (Table 3). The interaction between the gender and the diet only affected the FCR (*P* < 0.05), that is, the LF showed a higher FCR than the CF and the LM (*P* < 0.05 and *P* < 0.001, respectively). Finally, the LM tended to display a lower FCR than the CM (*P* = 0.058) (Figure 1). Regarding the weekly measurements, the treated groups displayed a higher LW than the controls from 42 days of age onward (*P* < 0.05), even if no significant differences were observed at 70 d of age (Figure 2). Moreover, improvements in the treated groups, compared to the controls, were recorded during the first 2 weeks of the trial (ADFI at 28–35 d and 35–42 d, *P* < 0.01 and *P* = 0.01, respectively; ADG and FCR at 35–42 d, *P* < 0.05; FCR at 28–35 d, *P* < 0.001, Supplementary Table S1). Further advantages were observed in the LM (ADG and FCR at 28–35 d, *P* < 0.05 and *P* < 0.001, respectively (Supplementary Figures S1, S2).

### Larvae consumption duration

**Figure 3:**
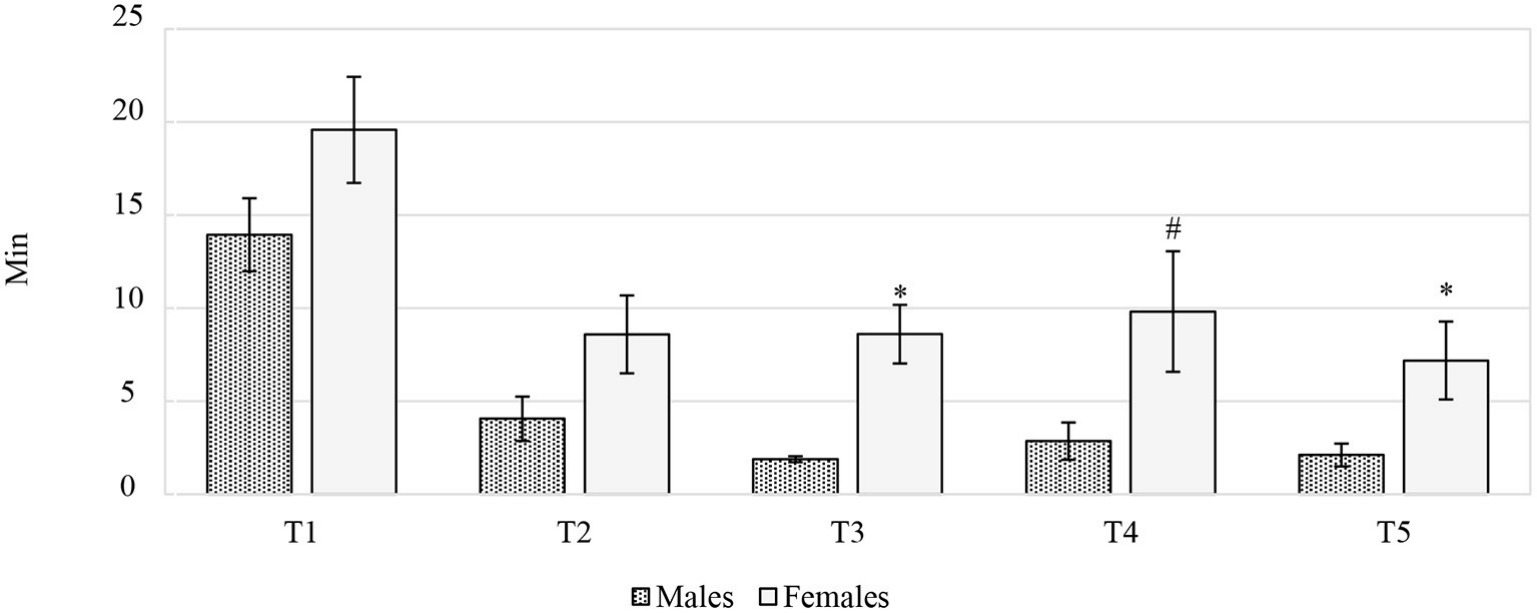
The time spent by the Label Rouge Naked Neck birds on eating live black soldier fly larvae (*n* = 10). *P ≤ 0.05; ^#^*P* ≤ 0.10. T1, 28–39 days of age; T2, 40–50 days of age; T3, 51–62 days of age; T4, 63–74 days of age; T5, 75–81 days of age.

The difference between males and females in the larvae consumption duration for each of the five recording periods is reported in Figure 3. Once the *in vivo* trial had been concluded, the real larvae intake was calculated. The study intended to provide the chickens with 10% live BSFL, on the basis of the expected daily feed intake, as reported by Hubbard (19). However, when the real feed intake was considered, BSFL ingestion was 10.41% and 8.01% for the F and M, respectively. Overall, the gender had a significant effect on the larvae consumption duration (min), which was lower in M than in F (4.97 vs. 10.76 min, *P* < 0.01). Furthermore, the age of the birds also had a significant effect on this parameter. The highest values were recorded at T1 for both sexes (T1:16.52, T2:5.90, T3:4.03, T4:5.29, T4:3.89 min, *P* < 0.001). The interaction between time and gender significantly influenced the larvae consumption duration (*P* < 0.01). In particular, significant differences were recorded between sexes at T3 and T5, while a statistical trend was observed at T4, when the F spent more time than the M on larvae consumption (*P* < 0.001, *P* < 0.05 and *P* = 0.072, respectively) (Figure 3). Moreover, the F ate larvae faster at T5 than at T4 (*P* < 0.05), but no significant differences were recorded between T5 and T2-T3. The M tended to consume the larvae faster at T2 than at T3 (*P* = 0.066), while a significant reduction in larvae consumption duration was observed between T2 and T5 (*P* < 0.05). Finally, the M tended to eat larvae faster at T5 than at T4 (*P* = 0.055) (data not shown).

### Slaughtering performance

**Figure 4:**
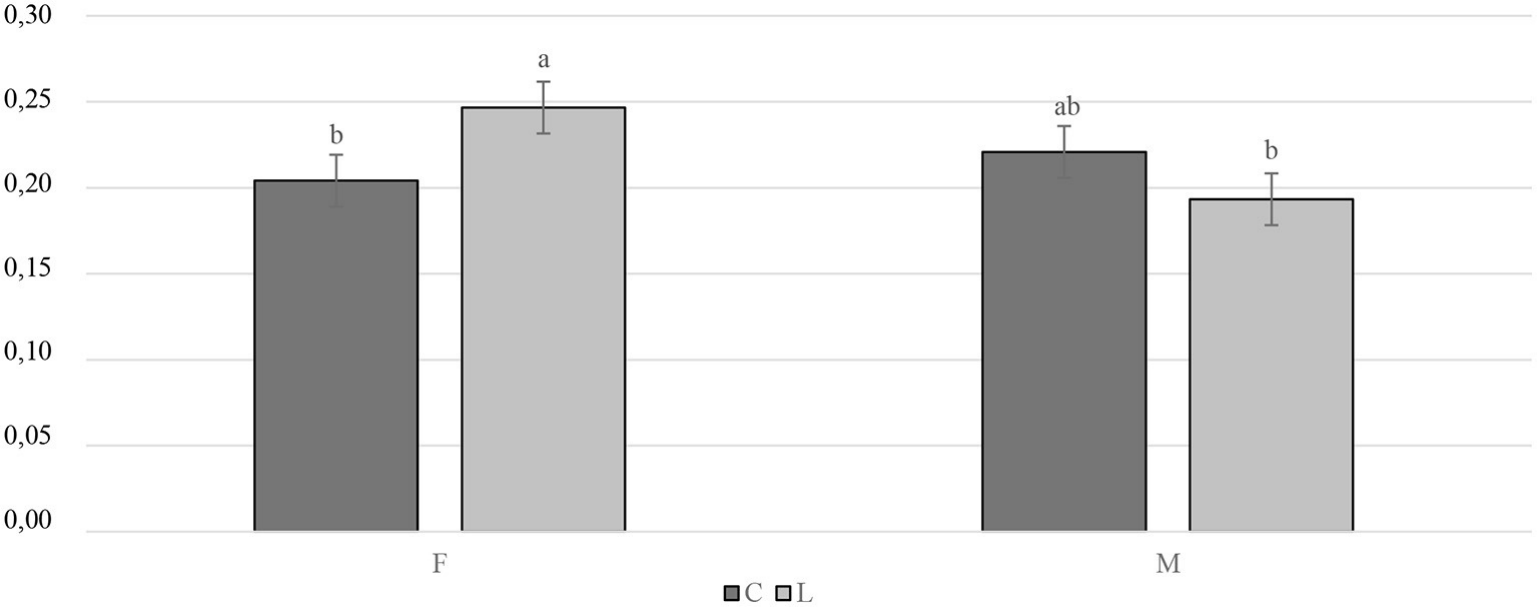
Interaction effect (gender × diet) on the bursa of Fabricius relative weight of the Label Rouge Naked Neck birds fed a diet supplemented with 10% live black soldier fly larvae; supplementation based on the expected daily feed intake, (28–81 d; *n* = 6). a, b indicate significant differences at *P* < 0.05; F, females; M, males; C, control; L, larvae.

The slaughtering performance is reported in Table 4. As expected, the SW (g) was influenced by the gender, with the M being heavier than the F (*P* < 0.001). No differences were observed in the CC yield (%LW) or in the RTCC yield (%LW) between the treated and the C groups, or in the RTCC yield (%LW). A trend was recorded for the gender, with the M tending to display a higher CC yield than the F (*P* = 0.091). The breast yield (%CC weight) was higher in the F than in the M (*P* < 0.001). On the other hand, the M showed a better thigh yield (%CC weight) than the F (*P* < 0.001). As far as the organ weights are concerned, the C groups showed a lower relative weight of the spleen (%SW) than the supplemented ones (*P* < 0.01). A statistical trend of the relative weight of the liver (%SW) was observed for gender and tended to be greater in the F than in the M (*P* = 0.087). The interaction between diet and gender had a significant impact on the relative weight of the bursa of Fabricius (%SW). Specifically, the LF groups displayed a higher relative weight of the bursa of Fabricius than the CF and the LM ones (*P* < 0.05) (Figure 4). The relative weight of the heart (%SW) was higher in the M than in the F (*P* = 0.001). Moreover, the diet tended to affect the relative weight of the heart (%SW), with the C groups which tended to show a lower value than the treated ones (*P* = 0.057). Finally, the relative weight of the abdominal fat (%SW) was greater in the F than in the M (*P* < 0.001) (Table 4).

**Table 4:** The slaughtering performance of the male and female Label Rouge Naked Neck birds fed a diet supplemented with 10% live black soldier fly larvae; supplementation based on the expected daily feed intake (28–81d; *n* = 12).

**Item**	**Diet (D)**		**SEM**	**Gender (G)**		**SEM**	***P-*** **value**		
	**BSFL**	**Control**		**Male**	**Female**		**D**	**G**	**D × G**
Slaughter weight (g)	2,441	2,423	10.00	2,829	2,035	10.00	0.206	<0.001	0.161
CC yield (%SW)	65.2	64.7	0.28	65.3	64.6	0.28	0.213	0.091	0.193
RTCC yield (%SW)	66.2	65.7	0.30	66.3	65.6	0.30	0.256	0.132	0.183
Breast yield (%CC)	23.2	23.2	0.21	22.2	24.2	0.21	0.815	<0.001	0.894
Thigh yield (%CC)	33.9	34.0	0.20	34.8	33.1	0.20	0.700	<0.001	0.953
Spleen (%SW)	0.16	0.14	0.00	0.14	0.15	0.00	0.002	0.150	1.000
Bursa of Fabricius (%SW)	0.22	0.21	0.01	0.21	0.23	0.01	0.619	0.224	0.020
Liver (%SW)	1.87	1.85	0.04	1.81	1.90	0.04	0.739	0.087	0.975
Heart (%SW)	0.51	0.48	0.01	0.52	0.47	0.01	0.057	0.001	0.771
Abdominal fat (%SW)	1.43	1.60	0.18	1.01	2.02	0.18	0.511	<0.001	0.270

SW, slaughter weight; CC, cold carcass; RTCC, ready to cook carcass; BSFL, black soldier fly larvae; SEM, standard error of the mean.

### Blood traits

The results about the blood traits of the birds are reported in Tables 5, 6. Overall, the live provision of BSFL did not impair most of the hematological traits or serum proteins and lipids, serum minerals, or liver and renal enzymes. As reported in Table 5, the leukocyte percentage was higher in the treated groups than in the C groups (*P* < 0.05). The eosinophil percentage was higher in the F than in the M (*P* < 0.01), whereas the opposite was found for monocytes (*P* < 0.05). Moreover, the C groups showed a higher monocyte percentage than the treated ones (*P* < 0.01). Regarding the serum lipids, the triglycerides tended to be more abundant in the F than in the M (*P* = 0.061), while cholesterol tended to be lower in the treated groups than in the C groups (*P* = 0.091). As for the serum minerals and the liver and renal enzymes, the live larvae supplementation only influenced the GGT (U/I), which was lower in the treated groups than in the C groups (*P* < 0.05) (Table 6). No significant effects were observed for the other blood parameters for either the fixed factors (gender and diet) or for the interaction between the gender and diet (*P* < 0.05).

**Table 5:** The hematological traits and serum proteins and lipids of the male and female Label Rouge Naked Neck birds fed a diet supplemented with 10% live black soldier fly larvae; supplementation based on the expected daily feed intake (28–81d; *n* = 12).

**Item**	**Diet (D)**		**SEM**	**Gender (G)**		**SEM**	***P-*** **value**		
	**BSFL**	**Control**		**Male**	**Female**		**D**	**G**	**D × G**
Erythrocytes, 10^6^, cell/μL	2.32	2.22	0.23	2.49	2.07	0.23	0.770	0.191	0.941
Leukocytes, 10^3^, cell/μL	31.1	23.9	2.26	26.7	27.2	2.24	0.023	0.745	0.955
Heterophils, %	47.3	42.1	2.90	47.2	42.2	2.90	0.202	0.225	0.976
Lymphocytes, %	48.1	52.8	2.65	48.2	52.8	2.65	0.212	0.218	0.955
Eosinophils, %	1.40	1.55	0.18	1.17	1.85	0.19	0.541	0.008	0.385
Monocytes, %	1.63	3.06	3.33	2.85	1.75	0.32	0.002	0.016	0.373
Basophils, %	2.90	2.67	0.32	2.54	3.05	0.32	0.617	0.269	0.495
Serum proteins and lipids
Total protein, g/dL	4.61	4.73	0.11	4.70	4.65	0.11	0.405	0.743	0.747
Cholesterol, mg/dL	107	119	5.14	116	109	5.14	0.091	0.345	0.520
Triglycerides, mg/dL	112	107	8.54	98.9	121	8.54	0.157	0.061	0.177

BSFL, black soldier fly larvae; SEM, standard error of the mean.

**Table 6:** The serum minerals and the liver and renal functions of the male and female Label Rouge Naked Neck birds fed a diet supplemented with 10% live black soldier fly larvae; supplementation based on the expected daily feed intake (28–81d; *n* = 12).

**Item**	**Diet (D)**		**SEM**	**Gender (G)**		**SEM**	***P-*** **value**		
	**BSFL**	**Control**		**Male**	**Female**		**D**	**G**	**D × G**
Liver function	
ALT, U/l	9.95	11.7	1.03	11.08	10.6	1.03	0.220	0.755	0.383
AST, U/l	167	145	10.27	160	152	10.27	0.134	0.606	0.079
GGT, U/l	22.8	26.9	1.13	26.1	23.6	1.13	0.011	0.114	0.792
Renal function	
Creatinine, mg/dL	0.46	0.47	0.03	0.49	0.44	0.03	0.811	0.148	0.561
Uric acid, mg/dL	8.94	8.95	0.59	8.81	9.07	0.59	0.986	0.752	0.936
Minerals	
P, mg/dL	8.97	9.14	0.31	8.85	9.26	0.31	0.695	0.349	0.294
Fe, μg/dL	101	94.2	17.76	85.5	109	17.76	0.785	0.336	0.300
Mg, mg/dL	1.22	1.10	0.23	1.04	1.28	0.23	0.696	0.450	0.796

BSFL, black soldier fly larvae; SEM, standard error of the mean; ALT, alanine-aminotransferase; AST, aspartate-aminotransferase; GGT, gamma-glutamyl transferase; CRE, creatinine; P, phosphorous; Fe, iron; Mg, magnesium.

## Discussion

### Growth performance

In the present study, the LW, ADG, and ADFI were higher, and the FCR was better in the M than in the F (*P* < 0.01). These results are in agreement with those reported in literature for different growth rates in both chicken hybrids and chicken breeds (30–32). The LW did not differ among the groups at the beginning of the trial, but it was higher in the treated birds than in the controls at the end of the rearing cycle. Interestingly, such differences were already visible at 42 days of age, thus also pointing out advantages for birds slaughtered early. However, the larvae were not included in the formulation of the diet, with a consequent difference in the average nutrient intake among L and C groups (CP 24.24 g/d, EE 6.52 g/d, and GE 2.00 MJ/d vs. CP 23.02 g/d, EE 5.62 g/d, and GE 1.94 MJ/d on an as fed basis, respectively). Therefore, the difference in the LW could be explained by the difference in the CP content of the diets. A higher final LW was reported at 5 weeks of age by Veldkamp and van Niekerk (13) in a trial conducted on turkeys fed 10% live BSFL. However, the results obtained in chicken experiments are inconstant. For instance, Bellezza Oddon et al. (15) observed no effects on the growth performance of broilers fed 5% live BSFL. Dabbou et al. (33) instead reported a higher LW at 10, 24 and 35 days of age in broiler M fed 10% BSFL defatted meal, while de Souza Vilela et al. (34) found an increase in the body weight of broilers fed 20% BSFL full-fat meal. Since live larvae contain around 70% of water (35), the nutrients of live larvae are more diluted than in a larvae meal, which might make the effect of live larvae less visible over brief periods. The overall FCR of the LF was higher than the CF and the LM (*P* < 0.05 and *P* < 0.001, respectively), while the LM tended to show a better FCR than the CM (*P* = 0.058), thus suggesting a beneficial use of live BSFL, albeit only for the M. However, the real percentage of larvae ingested on the basis of the DFI was 10.41% for the F and 8.01% for the M, although the expected DFI between the M and the F was identical (19) and, consequently, so were the grams of live larvae administered to the birds. Moreover, the observed ADFI differed between sexes, and the M displayed higher values than the F (*P* < 0.001). Therefore, the FCR results could be related to the different percentages of larvae provided, resulting in lower % of BSFL needed to obtain benefits in this parameter. However, once again, the published data are inconstant, and are mainly focused on insect meal studies. De Souza Vilela et al. (34) reported a decrease of 10% in the FCR of broilers administered 20% BSFL meal. Moreover, Gasco et al. (36) have stated that levels of BSFL meal of up to 15% are not disadvantageous for the growth of birds. No differences were reported for the weekly measurements of the growth performance, except at the beginning of the trial, where greater performances were observed for the supplemented birds (ADFI at 28–35 d and 35–42 d; ADG and FCR at 35–42 d) and mostly for the M (ADG and FCR at 28–35 d), thus suggesting an advantageous provision of live BSFL, especially for young M (Supplementary Table S1).

### Larvae consumption duration

Live insects are a natural source of food for chickens and their administration in rearing systems may represent an environmental enrichment and a positive stimulus for poultry behavior (6). The attractiveness of larvae is influenced by their motility, which draws the attention of chickens (9). The larvae consumption duration had previously been evaluated in trials conducted by Veldkamp and van Niekerk (13) and Bellezza Oddon et al. (15) in turkeys and broilers, respectively, but the inclusion of the gender as a fixed factor has never been considered before. The social behavior of birds might be involved. The pecking order is mainly established during the first weeks of age, and the thus created dominance relationships tend to be maintained, especially in F (37). As a consequence, the larvae consumption dynamics could be more constant in F, which could eat larvae calmer than M. The larvae consumption duration of the birds was significantly higher for both the M and the F at the beginning of the trial than at the other considered periods. Since an unknown object was introduced into the chicken pen, some time for adaptation and neophobia resolution was expected, as was its reduction at the end of the experiment. However, the time dedicated to larvae consumption at the end of the experiment (7.19 and 2.11 min for F and M, respectively) was not as low as that registered for turkeys (below 2 min) by Veldkamp and van Niekerk (13). Additionally, the larvae consumption duration reported for broilers (around 6.5 min; (15) was lower than the F consumption time of this trial, but higher than that of the M. Such differences are probably related to social behavior (37). Moreover, genetic selection could also affect the behavioral pattern of chickens (38, 39), which could explain the differences from the larvae consumption duration of the other studies. However, further investigations are required to confirm this hypothesis.

### Blood traits

Overall, the live administration of BSFL did not negatively influence the blood traits of the birds, as has already been reported in other studies conducted with both BSFL meal and live larvae in different poultry species (13, 16, 30, 57). The GGT was detected in lower concentrations in the supplemented groups than in the controls (*P* < 0.05). Since the GGT concentration in plasma represents an indicator of the health status of the liver, this result suggests a beneficial effect of live BSFL on this organ (40). The leukocyte concentration, as well as the monocyte one, was higher in the groups fed BSFL than in the controls (*P* < 0.05 and *P* < 0.01, respectively). However, contradictory results were obtained in previous studies, which did not report any significant differences related to meal inclusion or live BSFL provision (15, 16, 33, 41). On the other hand, de Souza Vilela et al. (34) reported a reduction in the white blood cells of broilers fed increasing levels (0.5, 10, 15, and 20%) of full-fat BSFL meal, thus suggesting a positive effect on the immune system. However, no definitive conclusion can be drawn on this section and further research is needed. Finally, eosinophils (%) were higher in the F than in the M, whereas the opposite was found for monocytes (%). Differences between sexes in chicken blood values have already been found. Peters et al. (42) reported generally lower mean values of the hematological parameters (red blood cell count, hemoglobin, packed cell volume, white blood cell count, mean corpuscular volume, mean corpuscular hemoglobin concentration, serum glucose, urea, cholesterol, albumin, globulin and creatinine) in F than in M, while Addass et al. (43) reported higher levels of white blood cells in F than in M, although such differences might vary among chicken genotypes (18). However, the hematological parameters in the present study are in line with those reported in previous studies conducted on chickens and ducks fed live insects and insect meals (15, 16, 33, 44). The triglycerides tended to be more abundant in the F than in the M (*P* = 0.061), although Whitehead and Griffin (45) and Musa et al. (46) did not observe any differences in the blood triglyceride level between sexes in broilers and two Chinese chicken breeds. However, a high level of triglycerides in the blood could lead to an increase in the abdominal fat deposition (47), according to what has been found in the current research, as the abdominal fat of the F was double that of the M (2.02 vs. 1.01%, respectively). The cholesterol tended to be lower in the treated groups than in the C groups. Similar results were found for laying hens (48, 49) and Muscovy ducks fed insect meal (16). This result is probably related to the chitin content of the larvae, which could bind the cholesterol and significantly reduce its presence in blood (50, 51).

### Slaughtering performance

Overall, the gender had a significant effect on the carcass traits of the birds, with M showing better values than F for the SW, and the thigh yield (%CC weight). However, the F showed a higher breast yield (%CC weight) than the M. Such differences, which are related to sexual dimorphism, were also reported in previous studies conducted on both broilers and local Italian chicken breeds (52–55). The relative weight of the spleen (%SW) was improved as a result of the supplementation of live BSFL and was heavier in the treated groups than in the controls. Similarly, the relative weight of the Bursa of Fabricius (%SW) was higher in the LF than in the CF and the LM. The weight of these organs, which are involved in the immune system response, could be considered as an indicator of the immunity system activity. Moreover, stressed birds generally show a reduction in the dimensions of their organs due to a corticosterone effect (49, 56). Considering these results, the provision of live BSFL could be beneficial for the immune system, probably because of its positive effect on bird welfare (improving birds' immune system reaction to stressors), as well as the larval chitin content, which has an immunostimulant effect (49, 57). Similar findings have been reported by Bellezza Oddon et al. (15) for broiler chickens. However, the bursa of Fabricius was only affected by the diet in the female group, thus suggesting a higher sensitivity to the chitin effect in F than in M. Indeed, Glick (58) reported that a physiological reduction of the bursa of Fabricius dimension occurred during chicken growth, which was more marked in M, due to the development of testicles and the related testosterone activity. The relative weight of the organs (%SW) in our study varied between sexes. The gender was responsible for a statistical trend on the relative weight of the liver that tended to be higher in the F than in the M, in a similarly way to what was reported by Mosca et al. (53) for the liver weight (g) in a study conducted on the Milanino chicken breed reared under free-range conditions. Benyi et al. (52) instead reported a heavier liver (g) in males than in female broiler chickens, whereas Olawumi et al. (59) found no differences in broilers. Moreover, the F displayed a heavier relative weight of the heart (%SW) than the M, although previous research reported no differences between gender for this parameter (52). Finally, the heart tended to be heavier in the treated groups than in the controls. Since no similar findings have been reported in previous articles related to insect supplementation, and inconsistent results have been provided in other studies, a univocal hypothesis cannot be drawn. For instance, Badmus et al. (60) observed an increase in the relative weight of the heart (% body weight) in broilers supplemented with dietary corticosterone at 4 and 6 weeks of age. Lin et al. (61) instead reported a decrease in the heart weight of corticosterone-supplemented broiler chickens after 7 days of administration, but not after 11, when compared to the control groups. Thus, the heart weight increase can be either negative or positive, and this aspect requires further investigation. The abdominal fat was more abundant in the F than in the M, thus confirming the previous results of Shahin and Elazeem (62) and Benyi et al. (52).

## Conclusions

This study has revealed, for the first time, the potential of the provision of live BSFL on the performance and blood traits of medium-growing chickens. In the study, such a supplementation did not undermine the performance or the health of the birds. Additionally, the LW of the chickens benefitted from this supplementation, as did the FCR, ADG, and ADFI of the young birds, with more advantages observed in males than in females. Moreover, such insects, likely because of their chitin content, might have a positive effect on the hepatic function, as they reduced the GGT concentration in the blood. Finally, the immune system was ameliorated by the provision of live BSFL, increasing the bursa of Fabricius and spleen dimensions. However, further investigations are needed to clarify the effects related to the provision of live BSFL to medium and slow-growing chicken genotypes.

## Data availability statement

The original contributions presented in the study are included in the article/Supplementary material, further inquiries can be directed to the corresponding author.

## Ethics statement

The animal study was reviewed and approved by BioEthical Committee of the University of Turin (Italy) *Via* Verdi 8, 10124, Turin (Italy).

## Author contributions

FG, AS, VB, MG, and IB designed the experiment. VB, MG, EC, and VZ took care of the animal rearing. CC provided the live black soldier fly larvae. VB and IB performed the statistical analysis. VB wrote the first draft of the manuscript. AS supervised the study. All authors carried out the *post-mortem* analyses, contributed to the creation of the manuscript, and approved the submitted version.
