# Supplementary material for: Black soldier fly larvae used for environmental enrichment purposes: Can they affect the growth, slaughter performance, and blood chemistry of medium-growing chickens?
Source: Front Vet Sci. 2022 Dec 14;9:1064017. doi: 10.3389/fvets.2022.1064017 (PMC9794612; doi:10.3389/fvets.2022.1064017)
Supplement: Supplementary file 1 [file Data_Sheet_1.pdf]

frontiers

Supplementary materials

Table S1. Weekly growth performance of males and females Label naked neck birds fed a diet supplemented with 10% live black soldier fly larvae supplementation based on the expected daily feed intake, (28-81d; n = 6).

Item	Days	Diet (D)		Gender (G)		SEM			*P*-value		
		BSFL	Control	Male	Female	D	G	D×G	D	G	D×G
LW g	28	479	475	515	439	2.07	2.07	2.92	0.186	<0.001	0.298
	35	692	683	757	618	3.93	3.93	5.56	0.082	<0.001	0.198
	42	939	924	1034	829	5.06	5.06	7.15	0.031	<0.001	0.150
	49	1207	1185	1343	1049	7.03	7.03	9.93	0.022	<0.001	0.527
	56	1498	1472	1684	1285	7.85	7.85	11.10	0.020	<0.001	0.989
	63	1772	1744	2006	1510	10.55	10.55	14.93	0.062	<0.001	0.759
	70	2040	2013	2333	1719	11.57	11.57	16.32	0.102	<0.001	0.325
	77	2244	2212	2584	1872	11.36	11.36	16.05	0.050	<0.001	0.868
	81	2372	2340	2742	1970	11.40	11.40	16.12	0.047	<0.001	0.281
ADG g/d	28-35	31	30.1	34.6	26.4	0.36	0.36	0.51	0.070	<0.001	0.008
	35-42	35.2	34.4	39.6	30.1	0.30	0.30	0.42	0.049	<0.001	0.286
	42-49	38.2	37.2	44.1	31.4	0.41	0.41	0.57	0.071	<0.001	0.320
	49-56	41.8	40.8	48.9	33.6	0.53	0.53	0.72	0.159	<0.001	0.730
	56-63	39.1	38.8	45.9	32.1	0.67	0.67	0.95	0.761	<0.001	0.476

	63-70	38.5	37.5	46.3	29.7	0.91	0.91	38.02	0.459	<0.001	0.426
	70-77	29.5	30.2	36.3	23.5	0.91	0.91	1.29	0.606	<0.001	0.853
	77-81	29.5	28.8	36.8	21.5	1.20	1.20	1.70	0.700	<0.001	0.254
ADFI g/d	28-35	60.6	63.7	70.1	54.2	0.68	0.68	0.96	0.002	<0.001	0.374
	35-42	78.1	80.6	89.4	69.2	0.71	0.71	1.00	0.010	<0.001	0.605
	42-49	95.1	97.3	108	83.6	1.05	1.05	1.48	0.139	<0.001	0.561
	49-56	105	108	122	91.5	0.99	0.99	1.40	0.069	<0.001	0.068
	56-63	124	126	138	111	1.40	1.40	1.98	0.350	<0.001	0.045
	63-70	135	133	152	116	1.44	1.44	2.03	0.493	<0.001	0.210
	70-77	153	150	164	139	2.40	2.40	2.40	0.426	<0.001	0.020
	77-81	144	146	163	126	3.32	3.32	4.69	0.654	<0.001	0.175
FCR g/g	28-35	1.85	2.04	2.00	1.89	0.04	0.04	0.05	<0.001	0.024	<0.001
	35-42	2.09	2.14	2.08	2.15	0.02	0.02	0.03	0.042	0.015	0.643
	42-49	2.34	2.39	2.27	2.46	0.02	0.02	0.03	0.073	<0.001	0.289
	49-56	2.38	2.41	2.28	2.51	0.02	0.02	0.03	0.474	<0.001	0.926
	56-63	3.02	2.95	2.79	3.18	0.04	0.04	0.06	0.278	<0.001	0.212
	63-70	3.26	3.41	3.06	3.61	0.06	0.06	0.08	0.063	<0.001	0.002
	70-77	5.09	4.67	4.20	5.55	0.16	0.16	0.23	0.067	<0.001	0.091

77-81	4.81	4.89	4.12	5.58	0.2	0.2	0.3	0.769	<0.001	0.472

Abbreviations: BSFL, black soldier fly larvae; LW, live weight; ADG, average daily gain; ADFI, average daily feed intake; FCR, feed conversion ratio (on a dry matter basis, including the larvae intake); SEM, standard error of the mean.

Figure S1. Interaction effect (gender×diet) on the feed conversion ratio at 28-35 days of age of the Label Rouge Naked Neck birds fed a diet supplemented with 10% live black soldier fly larvae; supplementation based on the expected daily feed intake, (28-81d; n = 6).

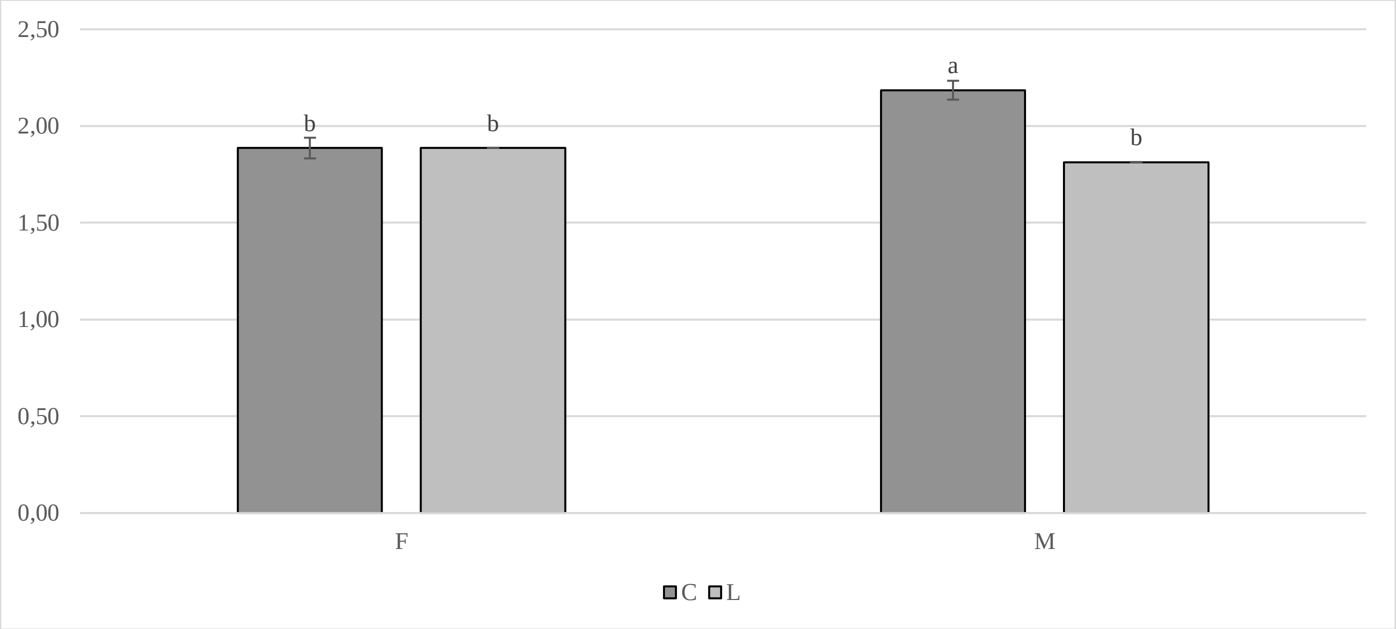

a,b indicates significant differences among groups, P<0.05. F: females; M: males; C: control; L: larvae.

Figure S2. Interaction effect (gender×diet) on the average daily gain at 28-35 days of age of the Label Rouge Naked Neck birds fed a diet supplemented with 10% live black soldier fly larvae; supplementation based on the expected daily feed intake, (28-81d; n = 6).

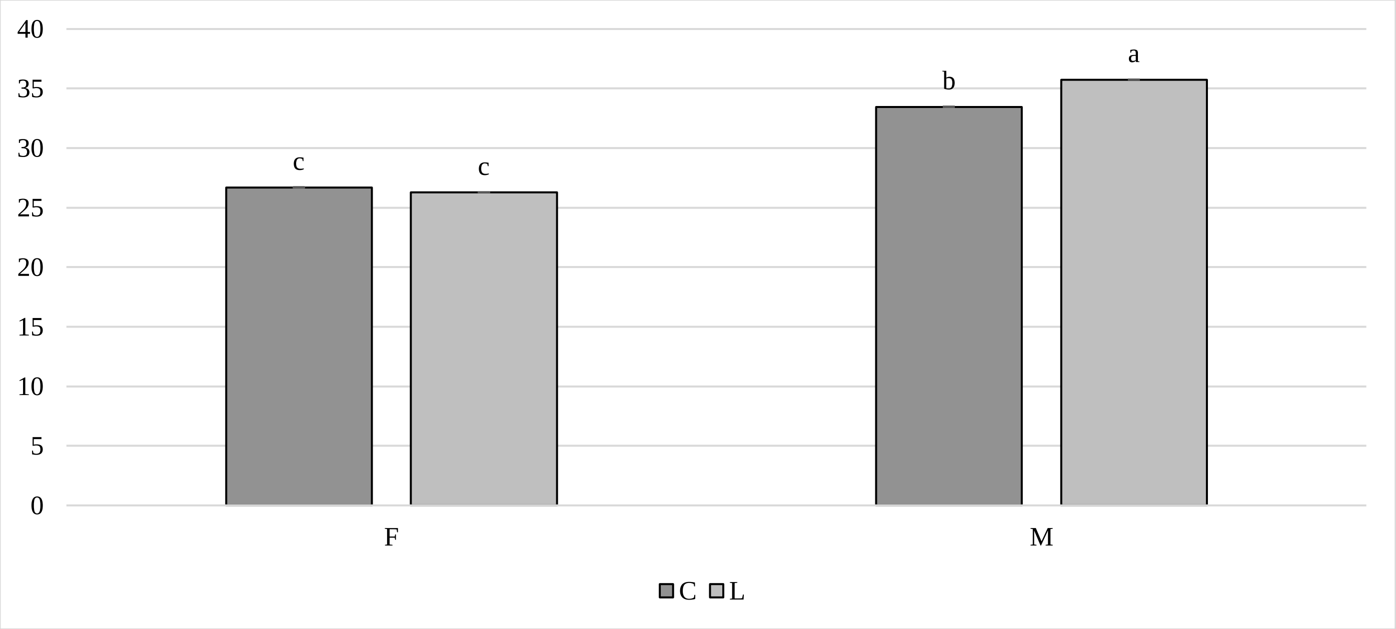

a,b,c indicates significant differences among groups, P<0.05. F: females; M: males; C: control; L: larvae.
